# Phase 2 non-randomised trial of secondary cytoreduction and hyperthermic intraperitoneal chemotherapy in recurrent platinum-sensitive ovarian cancer

**DOI:** 10.3332/ecancer.2021.1260

**Published:** 2021-07-05

**Authors:** Hemanth Raj, Marri Sri Santosh Keerthi, Ravisankar Palaniappan, Ujwala Prakash, Manikandan Dhanushkodi, Trivadi S Ganesan

**Affiliations:** 1 Department of Surgical Oncology, Cancer Institute (WIA), 38, Sardar Patel Road, Chennai 600036, Tamil Nadu, India; 2 Department of Surgical Oncology, Sri Venkateshwaraa Medical College Hospital and Research Centre, Puducherry 605102, India; 3 Department of Medical Oncology, Cancer Institute (WIA), 38, Sardar Patel Road, Chennai 600036, Tamil Nadu, India; † Hemanth Raj and Marri Sri Santosh Keerthi contributed equally.; a https://orcid.org/0000-0002-8192-3856

**Keywords:** HIPEC, platinum-sensitive, recurrent ovarian cancer, secondary cytoreduction

## Abstract

**Background:**

The role of secondary cytoreduction with hyperthermic intraperitoneal chemotherapy (HIPEC) is not clearly defined in recurrent platinum-sensitive ovarian cancer (PSOC). There is a paucity of studies on secondary cytoreduction with HIPEC in PSOC from developing countries like India. This study was done to assess the feasibility and safety of secondary cytoreduction and HIPEC in recurrent PSOC.

**Methods:**

This was a prospective, non-randomised, open-label, phase 2 trial of secondary cytoreduction and HIPEC (Cisplatin 75 mg/m2 43°C over 60 minutes) in patients with recurrent platinum-sensitive epithelial carcinoma of ovary/fallopian tube/peritoneum done in a tertiary cancer centre from February 2016 to August 2019. The primary outcome was to assess the overall survival (OS) and the secondary outcomes were to assess the progression-free survival (PFS) and toxicity.

**Results:**

Twenty-seven patients were screened and among them, 15 patients were included in this analysis with a median follow-up of 25 months. The mean cancer antigen (CA) 125 at the time of recurrence was 149 U/mL (range: 10–2,030 U/mL) and the median platinum-free interval was 21 months. The perioperative chemotherapy used was paclitaxel + carboplatin 53.3% (8/15), liposomal doxorubicin + carboplatin 40% (6/15) and none 6.5% (1/15). The median Peritoneal Carcinomatosis Index score was 8 (range: 3–25). The Clavien Dindo score was I, II and III in 6.7%, 26.7% and 13.3% patients, respectively. Recurrence was radiological and biochemical in 60% (9/15) and 7% (1/15), respectively. The most common site of recurrence was intra-abdominal (peritoneal). The median PFS and OS were 15 months (range: 0–34) and 26 months (range: 23–29), respectively. The grade 3 or 4 toxicity was 40%.

**Conclusion:**

Secondary cytoreduction with HIPEC is feasible and safe in recurrent PSOC. Conclusive evidence that secondary cytoreduction with HIPEC is essential awaits the results from ongoing randomised controlled trials.

## Background

Epithelial ovarian carcinoma (EOC) being an indolent disease is often diagnosed at an advanced stage. The standard treatment for advanced ovarian carcinoma is primary cytoreduction followed by adjuvant platinum-based doublet chemotherapy. Despite being chemo-sensitive, 70% of patients have a recurrence during the first 3 years of follow-up with a dismal 5-year overall survival (OS) of 20%–30% [1]. The peritoneum is the most common site of recurrence in advanced ovarian cancer [2].

Cytoreduction with hyperthermic intraperitoneal chemotherapy** (**HIPEC) has been shown to improve survival in first line treatment of advanced ovarian cancer [3]. In patients with recurrent disease, secondary cytoreduction has been shown to improve survival based on many retrospective studies [4]. Factors (Arbeitsgemeinschaft Gynaekologische Onkologie (AGO) criteria) associated with improved survival after secondary cytoreduction are Eastern Cooperative Oncology Group (ECOG) performance status 0, absence of ascites > 500 mL, Federation of Gynecology and Obstetrics (FIGO) stage I and II at diagnosis and no residual tumour after primary surgery [5]. A phase 3 trial of secondary cytoreduction in platinum-sensitive ovarian cancer (PSOC) based on the AGO score has been shown to improve progression-free survival (PFS) by 5 months [6]. However, another phase 3 randomised controlled study [Gynecologic Oncology Group (GOG) 213] has shown that secondary cytoreduction does not improve PFS and OS in platinum-sensitive recurrent ovarian cancer [7]. Thus, the role of secondary cytoreduction in recurrent EOC remains uncertain.

There are several reports in the literature evaluating the role of HIPEC following surgery for recurrent ovarian cancer. There are at least two reports from Italy [8, 9] showing improvement in survival in recurrent disease. In one report, the median PFS was 27 months, while OS was 70 months. Similarly, a randomised trial from Greece [10] showed the HIPEC with maximal cytoreductive surgery (CRS) improved survival over surgery alone. Currently, there are at least two ongoing randomised trials (HORSE and CHIPOR) evaluating the role of HIPEC in recurrent ovarian cancer [11].

In India, there have been reports evaluating the role of HIPEC in peritoneal carcinomatosis that is amenable to surgery [12–14]]. However, there have been no randomised trials as randomised phase 3 trials will need many patients that need multicentre collaboration.

This study was done to assess the feasibility and safety of secondary cytoreduction and HIPEC in recurrent PSOC.

## Methods

### Participants & settings

This was a prospective, non-randomised, open-label, phase 2 trial of secondary cytoreduction and HIPEC in patients with platinum-sensitive recurrent epithelial carcinoma of ovary/fallopian tube/peritoneum done in a tertiary cancer centre from February 2016 to August 2019.

The inclusion criteria were consecutive recurrent PSOC (platinum-free interval > 6 months), age > 18 years, ECOG performance status < 2, absolute neutrophil count > 1,500/mm^3^, platelets > 1,50,000/mm^3^, international normalised ratio < 1.5, serum total bilirubin < 1.5 mg/dL, serum alkaline phosphatase < 2.5 times the upper limit of normal, normal renal functions and disease amenable for secondary cytoreduction. The exclusion criteria were patients with bowel obstruction, absence of peritoneal disease and non-epithelial/borderline ovarian cancer.

### Interventions

The preoperative evaluation included serum cancer antigen (CA) 125, chest X-ray and contrast-enhanced computed tomography (CECT) of abdomen/pelvis. All patients underwent planned perioperative chemotherapy for a total of six cycles using carboplatin-based doublet (paclitaxel/liposomal doxorubicin).

The surgery performed was secondary cytoreduction including resection of the recurrent tumour along with the peritoneum (including central peritoneal compartment, greater omentectomy, splenectomy, peritonectomy beneath both hemi-diaphragms, removal of tumour from the liver surface and pelvic peritonectomy). Bowel resection and anastomosis were done as required and intra-abdominal drains were placed. Temporary colostomy was done in cases where rectum was resected. Peritoneal Carcinomatosis Index (PCI) and completeness of cytoreduction scores were recorded. The skin incision was closed after placing inflow and outflow catheters for administering HIPEC.

HIPEC was delivered using EXIPER M03223, Menfis division, MEDICA S.P.A, Italy till February 2018 and thereafter using Performer HT, RanD, Medolla, Italy. The perfusion circuit consisted of two inflow catheters placed in the upper abdomen, two outflow catheters placed in the pelvis, a roller pump and a heat exchanger. Temperature probes were attached to inflow and outflow catheters. The premedication before HIPEC with cisplatin was injection (Inj) fosaprepitant 150 mg and Inj. palonosetron 0.25 mg intravenously, 30 minutes before cisplatin. Cisplatin was infused at a rate of 75 mg/m^2^ in 2 L/m^2^ peritoneal dialysis fluid at a temperature of 43°C over 60 minutes after attaining the minimum temperature of 42°C throughout the abdominal cavity, with a flow rate of 1,000 mL/minute.

During the post-operative period, patients were not fed orally till Ryle’s tube aspiration was <100 mL. Patients received antibiotics (Inj. cefuroxime 1,500 mg IV and Inj. metronidazole 500 mg IV for 3 days), proton pump inhibitors, prophylaxis for deep vein thrombosis (low molecular weight heparin, pneumatic calf pump) and analgesics. Abdominal drains were removed when the output was <50 mL. Patients were discharged when they were on a normal diet, drains removed, wound healed and sutures removed. The patients were followed up every 3 monthly for the 2 years, 6 monthly in the 3rd and 4th year and annually thereafter. During follow-up, patients underwent history, clinical examination and serum CA-125. CECT of abdomen/pelvis was performed in patients with rising CA-125 or when clinically indicated. The patient demographics, pre-operative investigations, intra-operative findings, complications (intraoperative and postoperative) and follow-up data were recorded.

### Statistical analysis

The PFS was calculated from the date of HIPEC until the date of progression or date of death or date of last follow-up. The OS was calculated from the date of HIPEC until the date of death or date of last follow-up. The survival was estimated using the Kaplan–Meier method. Data were analysed using SPSS, version 23 (SPSS Inc., IL, USA) and *p*-value of <0.05 was considered significant.

Written informed consent was taken from all the patients. The study was conducted according to various guidelines for the ethical conduct of studies including the good clinical practice guidelines, and the Indian Council of Medical Research. This study was in accordance with the ethical standards of the responsible committee on human experimentation (institutional or regional) and with the Helsinki Declaration of 1964, as revised in 2013. This trial was approved by Drug Controller General of India (DCGI), Scientific Advisory Committee (SAC), Institutional Ethics Committee (IEC) and registered in Clinical Trials Registry of India (CTRI).

## Results

**Figure 1.**
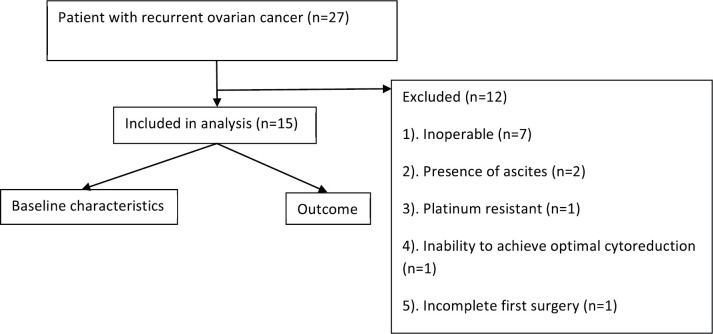
Study flowchart.

Twenty-seven patients (Figure 1) underwent screening among whom twelve were excluded from the trial. The reasons for exclusion were incomplete first surgery (*n* = 1), inoperable disease (*n* = 7), presence of ascites (*n* = 2), platinum-resistant disease (*n* = 1) and inability to achieve optimal cytoreduction (*n* = 1). Fifteen patients were included in this trial with a median follow-up of 25 months (Table 1). The median age was 51 years (range: 42–70 years). Co-morbid illness was present in 53% (8/15), the most common being diabetes (40%) followed by hypertension (13%). A family history of cancer was present in 26% (4/15), and the most common malignancy was breast cancer (13%). About 53% were postmenopausal and the rest were premenopausal, all women were ECOG performance status 1.

The stage at the initial diagnosis of ovarian carcinoma was III (80%) or IV (20%). The histology was serous adenocarcinoma, poorly differentiated adenocarcinoma and clear cell carcinoma in 73%, 20% and 7%, respectively. The differentiation was low to intermediate in 7% and high grade in 93%. The median CA 125 at presentation was 2,833 U/mL (range: 15–9,730 U/mL). All patients received neoadjuvant chemotherapy. The majority (93%, *n* = 14) received paclitaxel and carboplatin and one patient (7%) received liposomal doxorubicin and carboplatin. Pathological complete response was achieved in one patient (7%). All patients underwent optimal interval cytoreduction. After completion of six cycles of paclitaxel carboplatin, 73% of patients received one cycle of intraperitoneal cisplatin (100 mg). The median duration of remission after completion of all treatment was 22 months (range: 8–76 months).

The mean CA 125 at the time of recurrence was 149 U/mL (range: 10–2,030 U/mL) and the median platinum-free interval was 21 months. The perioperative chemotherapy used was paclitaxel + carboplatin 53.3% (8/15), liposomal doxorubicin + carboplatin 40% (6/15) and none 6.5% (1/15). The median PCI score before secondary cytoreduction was 8 (range: 3–25). The mean duration of surgery (secondary cytoreduction and HIPEC) was 455 + 85 minutes (Table 2). Following surgery, blood component support was required in 60% (9/15), total parenteral nutrition (TPN) in 87% (13/15), inotropes in 80% (12/15) and mechanical ventilation in 47% (7/12) patients. The median time to ambulation was the 4th postoperative day. The median duration of hospital stay was 13 days (range: 8–18). The nadir white blood cell count was 3,500 cells/mm^3^ and none of them had nephrotoxicity. The Clavien Dindo score was I, II and III in 6.7%, 26.7% and 13.3% patients, respectively.

**Figure 2.**
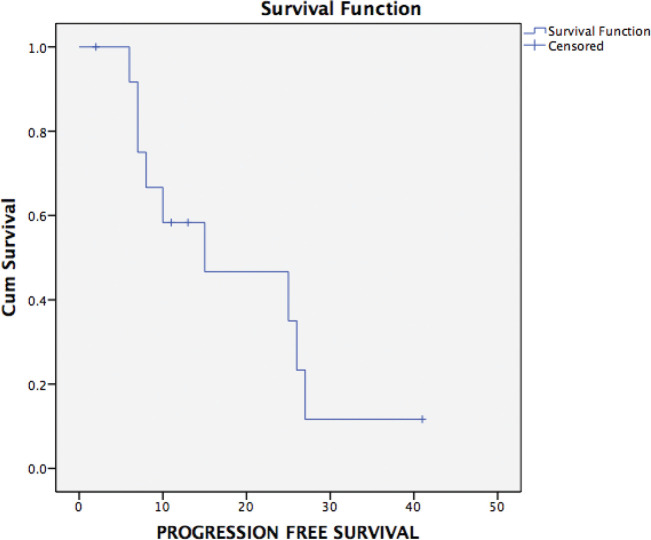
Kaplan–Meier analysis showing PFS of 13 patients with platinum sensitive recurrent ovarian cancer who underwent secondary cytoreduction with HIPEC. The median PFS was 15 months (range: 1–34 months).

**Figure 3.**
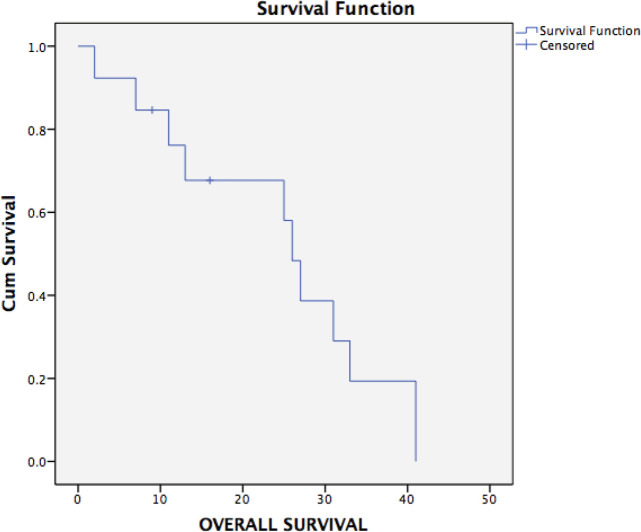
Kaplan–Meier analysis showing OS of 13 patients with platinum sensitive recurrent ovarian cancer who underwent secondary cytoreduction with HIPEC. The median OS was 26 months (range: 22.9–29.0).

Ten patients have had a recurrence on follow-up. Recurrence was radiological and biochemical in 60% (9/15) and 7% (1/15), respectively. The most common site of recurrence after secondary cytoreduction and HIPEC was intra-abdominal (peritoneal). Thirteen patients were evaluable for survival analysis. The median PFS (Figure 2) and OS (Figure 3) were 15 months (range: 1–34) and 26 months (range: 23–29), respectively. The most common systemic therapy for recurrent disease after secondary cytoreduction and HIPEC was liposomal doxorubicin carboplatin followed by oral etoposide. At this time of follow-up, two patients have died due to progressive disease.

## Discussion

The rationale for using HIPEC in ovarian cancer is that peritoneum is the most common site of recurrence, and HIPEC increases the drug concentration in the peritoneal cavity [15]. Moreover, hyperthermia causes direct cytotoxicity to cancer cells and is synergistic with chemotherapy [16]. Also, secondary CRS acts synergistically by reducing the bulk of the disease and thereby improving drug penetration and reducing the risk of further chemo-resistance.

The morbidity of the combined approach ranges from 20% to 66% and has been largely attributed to the extensive surgery required in an already previously operated patient. In the present study, the incidence of grade II complications and more was 40% as seen in other studies. The median duration of stay after cytoreduction and HIPEC of 8–25 days was previously reported which is also similar to our trial (median: 13 days, range: 8–18 days) [17].

The burden of peritoneal carcinomatosis determined by the PCI score [18] and the extent of cytoreduction achieved significantly influence the survival rates. Some studies have used PCI scores in identifying patients with limited peritoneal cancer indicating that those with a lesser PCI score could benefit more with the combined approach. A Chinese study of 40 patients showed that patients with PCI < 20 had better OS than those with PCI > 20 (76.6 versus 38.5 months, *p* = 0.01) [19]. In this trial, the median PCI was 8 (range: 3–25) which shows that our subset of patients could benefit more with CRS and HIPEC. Mulier *et al* [20] reported that complete cytoreduction was associated with a median OS and 5-year OS of 97.4 months and 63%–67%, respectively.

A phase 3 trial of 120 patients from Greece showed that cytoreduction with HIPEC improved survival as compared to only cytoreduction in patients with recurrent ovarian cancer. However, this study was criticised for inappropriate patient selection and statistical analysis [10]. A multicentre French study of 566 patients with advanced ovarian cancer showed improved survival with cytoreduction and HIPEC [21]. An Italian study with 12 patients showed that tertiary cytoreduction with HIPEC repetition is feasible [22].

The systemic therapy that has improved PFS in PSOC includes targeted therapy (bevacizumab) [23] based chemotherapy, Poly ADP Ribose Polymerase (PARP) inhibitors like olaparib [24], niraparib [25] and rucaparib [26]. Immunotherapy with pembrolizumab is an option in patients with microsatellite instability-high or mismatch repair deficient tumours [27]. In this trial, the median PFS and OS were 15 months and 26 months, respectively.

The strengths of this study are the prospective design and the first HIPEC study in Indian patients with recurrent ovarian cancer. The limitations include small sample size, single institutional study, non-randomised design, lack of breast cancer status and non-usage of targeted therapy (bevacizumab) and PARP inhibitors. The results of the phase 3 randomised controlled trials will throw more light on the efficacy of secondary cytoreduction and HIPEC.

## Conclusion

Secondary cytoreduction with HIPEC is feasible and safe in recurrent PSOC. Conclusive evidence that secondary cytoreduction with HIPEC is essential awaits the results from ongoing randomised controlled trials.

## Funding

This trial was supported by Cancer Institute (WIA), Chennai.

## Conflicts of interest

The authors declare that they have no conflicts of interest.

## Authors’ contributions

Conception: Hemanth Raj, Ravisankar Palaniappan, Trivadi S Ganesan. Acquisition: Marri Sri Santosh Keerthi, Hemanth Raj, Ujwala Prakash, Manikandan Dhanushkodi. Analysis: Marri Sri Santosh Keerthi, Manikandan Dhanushkodi, Trivadi S Ganesan. All authors made substantial contributions towards drafting and final approval, and they agree to be accountable on all aspects of the manuscript.

## Affiliations

None.

## Details of earlier presentation

None.

## Institutional review board approval

All procedures performed in studies involving human participants were in accordance with the ethical standards of the IEC and with the 1964 Helsinki Declaration and its later amendments or comparable ethical standards. The study was approved by the SAC on 03/01/2015, IEC on 21/02/2015, DCGI on 29/01/16 and CTRI on 04/09/2017.

## Figures and Tables

**Table 1.** Baseline characteristics.

**Demographics (*n* = 15)**		
Age in years	Median (range)	51 (42–70)
Co-morbid illness	Absent/present	8 (53%)/7 (47%)
Family history of malignancy	Absent/present	11 (73.3%)/4 (26.7%)
Menopausal status	Pre-menopausal/post-menopausal	7 (47%)/8 (53%)
**Variables at initial diagnosis (*n* = 15)**		
CA-125 at diagnosis (U/mL)	Median (range)	2833 (15.1–9,730)
Stage at diagnosis	III/IV	12 (80%)/3 (20%)
Tumour histology	Serous cystadenocarcinoma/clear cell carcinoma/poorly differentiated adenocarcinoma	11 (73.3%)/1 (6.7%)/3 (20%)
Tumour grade	Low or intermediate/high grade	1 (6.7%)/14 (93.3%)
Intravenous chemotherapy	Paclitaxel + carboplatin/others	14 (93.3%)/1 (6.7%)
Intra-peritoneal chemotherapy	Received	11 (73%)
**Variables at relapse / recurrence (*n* = 15)**		
CA-125 at relapse (U/mL)	Median (range)	149 (10.5–2,030)
Chemotherapy type	Paclitaxel + carboplatin/liposomal doxorubicin + carboplatin/none	8 (53.3%)/6 (40%)/1 (6.7%)
PCI score	Median (range)	8 (3–25)

**Table 2.** Treatment, toxicity & survival (*n* = 15).

Duration of surgery (minutes)	Mean ± 2 SD	455 ± 85
Grade of toxicity^a^	I/II	11 (73.3%)/4 (26.7%)
Blood transfusion		9 (60%)
Intra-operative hypotension		12 (80%)
Post-operative ventilator support		7 (46.7%)
Post-operative TPN		13 (86.7%)
Duration of post-operative stay (days)	Median (range)	13 (8–18)
Clavien Dindo score	0/I/II/III	8 (53.3%)/1 (6.7%)/4 (26.7%)/2 (13.3%)
Peri-operative chemotherapy	Received/not received	9 (60%)/6 (40%)
**Follow-up** (*n* = 13)		
Relapse		10 (76.9%)
Mortality	Alive/expired	11 (85%)/2 (15%)
PFS in months^b^	Median (range)	15 (1–34.5)
OS in months^b^	Median (range)	26 (22.9–29.0)

a According to the Common Terminology Criteria for Adverse Events

b Calculated using Kaplan–Meir analysis

SD, Standard deviation; TPN, Total parenteral nutrition
